# Doing ethics with microbes: toward a queer feminist posthuman framework for bioethics

**DOI:** 10.12688/wellcomeopenres.21391.1

**Published:** 2024-09-03

**Authors:** Tiia Sudenkaarne, Salla Sariola, The Centre for the Social Study of Microbes (CSSM)

**Affiliations:** 1 Sociology/CSSM, University of Helsinki, Helsinki, Uusimaa, Finland

**Keywords:** Social study of microbes, queer bioethics, feminist bioethics, more-than-human, justice, vulnerability, care

## Abstract

The Centre for the Social Study of Microbes (CSSM) at University of Helsinki, Finland is a Wellcome-funded hub for creating new approaches to human-microbial relations. Most urgently, the complex relations between microbes, antimicrobial resistance (in its human-health centered definition, understood as infection and disease now uncurable by antibiotics, threatening human life and wellbeing), animals, environments and climate emergency require new theoretical and methodological approaches. These include a variety of research interest from global fermentation practices and cultures to microbial bioremediation techniques, from aquatic microbes to urban gardens. A key orientation of CSSM is also combining ethnography with artistic and performative practice such as felting, painting, installation, sculpture and audiovisual means, to gain a deeper, more sensory and embodied perspective of our shared lives with microbes. As their agency exists outside textuality, new experimental methodology is needed to engage with it.

Thinking with microbes invites many ethical issues that often remain unaddressed in medical and scientific approach. As one of such contributions, we suggest a new framework for bioethics. In a conceptual analysis, a queer feminist posthuman framework aims for radical reorientation of human exceptionalism for more-than-human justice while keeping existing social justice issues between groups of people, such as vulnerabilities cascading around gender and sexual variance, in the same framework. Both a theoretical and practical initiative, seeking to forge solidarity between justice movements, this framework could ground policies relevant to a broader bioethical and philosophical research community, and social scientists studying microbes. This open letter discusses this work at the CSSM.

## Plain Language Summary

The Centre for the Social Study of Microbes (CSSM) at University of Helsinki, Finland is a Wellcome-funded hub for creating new approaches to human-microbial relations. These include a variety of research interest from global fermentation practices and cultures – the arts of rice beer and sake-making, for example – to microbial bioremediation techniques, from aquatic microbes to urban gardens. A key orientation of CSSM is also to explore microbes through artistic mediums such as felting, painting, installation, sculpture and audiovisual means.

Thinking with microbes invites many ethical issues that often remain unaddressed in medical and scientific approach. Most urgently, the complex relations between microbes, antimicrobial resistance (in its human-health centered definition, understood as infection and disease now uncurable by antibiotics, threatening human life and wellbeing), animals, environments and climate emergency require new ways to think about these issues and aims to resolve them. As one of such contributions, we suggest a new framework for bioethics. Sudenkaarne's queer feminist posthuman framework for bioethics offers a platform to consider injustices caused by human action to environments and more-than-human animals and injustices between groups of people in the same framework. Hopefully, this framework is inspirational to other researchers and public policy initiatives. It has been inspired by the collective work at CSSM.

## Disclaimer

The views expressed in this article are those of the author(s). Publication in Wellcome Open Research does not imply endorsement by Wellcome.

## Introduction

The rationale of this open letter is to discuss social scientific work with microbes from an applied ethics and moral theoretical point of view, which is a part of a philosophical approach to bioethics (e.g. Holland, 2016; O’Malley, 2014). In this approach, bioethics can be defined as considerations of the moral, societal, and political issues brought about by sickness, health, care, and environment: what kind of ontological assumptions these concepts build on, what kind of knowledges they entail and how should ethical issues around them be resolved. This notion of bioethics stands in stark contrast to perfunctory management of ethics as avoiding liability or purely a matter of conducting research ( Donchin & Purdy, 1999; Wahlert & Fiester, 2014; Wolf, 1996). Bioethical concepts must be situated in a specific ethical framework for them to do justice to the most “morally vulnerable” ( Fiester, 2015). 

This letter builds Sudenkaarne’s conference presentation at the Oxford Global Health and Bioethics 2023, in which she discussed how antimicrobial resistance (AMR) shows the limits of the existing challenges of the most dominant justice framework, and how could a queer feminist posthuman framework offer solutions to crisis of justice. Her work is situated in the research environment of the Centre for the Social Study of Microbes (CSSM) directed by Sariola. Sudenkaarne and Sariola are currently collaborating on applications of the framework. ^i^ In this letter, the focus is on the need for a new framework and the structure of this framework to grapple with ethical questions of AMR. 

In a conceptual analysis combining bioethical principlism, analytical philosophy, feminist thought and queer theory, Sudenkaarne introduces a queer feminist posthuman framework for justice. In this radical reorientation of human exceptionalism, the framework aims to question anthropocentrism. Sudenkaarne and Sariola have in previous collaborations respectively, offered considerations into more-than-human orientations, ( Cañada *et al.*, 2022; Sudenkaarne & Butcher, 2024) while seeking to keep existing social justice issues between groups of people in the same framework. In the case of AMR, to us this means analyzing how the scale of microbes ‘sticks’ to some bodies more than others based on particular sexual practices and maps on to particular bodies and orifices: vulnerability of same-sex relations, in the global south in particular.

The purpose of this letter is to introduce both a theoretical and practical initiative that seeks to further ground solidarity between justice movements while simultaneously promoting a more-than-human-justice orientation for cosmopolitical flourishing. It also aims to offer this framework as relevant to a broader bioethical, social scientists working on human-microbial relations, and philosophical research community and for grounding policy. This includes acknowledging how more-than-human actors already play an important role in environmental and climate politics ( Sudenkaarne & Butcher, 2024). Furthermore, framework-thinking is crucial for AMR as, most recently noted by Pokharel *et al*. (2024), efforts to both conceptualize and address AMR are challenged by ethical tensions. To us, ethical tensions between different actors in AMR require a new framework that does not silo between humans, more-than-human animals and environments but understand them as compounded, suggesting post-anthropocentric solutions to AMR. New moral theories and ethical frameworks are needed to achieve this. Before discussing these tensions further, however, we will discuss this work as part of the more-than-human community of the CSSM.

## Background: making ethics in a slime mold that is the CSSM

Enhancing more-than-human thinking in social study of microbes is the driving vision for the CSSM. As envisioned by director Sariola and CSSM co-investigators Jose Cañada and Mikko Jauho in crafting the field of social study of microbes, the global proliferation of AMR, climate emergency (CE), and now the COVID-19 global pandemic are examples of the urgency to find new ways of living in a more sustainable and socially just way with each other, with other animals, plants and our microbial ancestors. The collective aim of research at the CSSM is to create novel theorization for the analysis of and paradigm shift in human-animal-microbial relationships. From the global governance of AMR and practices with microbial materials; through biotechnical practices with humans and microbes; to traditional and contemporary fermentation practices, our research projects and practices are interdisciplinary, geographically dispersed, and multi-scalar.

A research community coming together from various intellectual traditions, from analytical philosophy to science and technology studies, from arts to natural sciences, CSSM's collective aim is to develop novel theories and methodologies for producing knowledge about and with microbes.

CSSM’s work builds on the premise that microbes are not only biological entities but also shape, and are shaped by, our social worlds. Doing ethics with microbes means taking on the challenges they pose to practical ethics -- for example, how to address the scale of microbes that ‘sticks’ to some bodies more than others based on particular sexual practices, or the global complexities of access versus excess of antibiotics -- but also to moral theory to conceptualize the complex relationality between human and more-than-human. Further, it requires formulating relational ontologies, including posthuman perspectives to a global public health ( Cohn & Lynch, 2017) and how posthumanist approaches can illuminate current issues in bioethics ( Sands, 2022). 

To alleviate these ethical challenges more sustainably, new ways of being, sensing, doing and knowing with microbes are required. This calls for new, experimental methods breaking away from hierarchical knowledge-production. The ambitious aim of paradigm shift is crucially dependent on collaboration. Thus the CSSM is an exploratory setting in which new non-hierarchical collaborative practices are developed, seeking inspiration from how the slime mold evolves without a central organizing body ( Hey *et al.*, 2023). This has led to fruitful anarchy in a flat organization without central order but not without leadership and responsibility. This unruliness to be further explored by us as a philosophical concept elsewhere, is a key source for intellectual creativity. We have formulated, in an open-ended dynamic process, our own safe space practices building on the principles of care and respect of both diversity and biodiversity. Under the auspices of CSSM, we commit to being open, aware, and reflexive of power dynamics pertaining to knowledge production and actively seek to undo them. These systems of oppression that we see underlying academia are specific to university as well as society more generally. The reflexivity includes consideration of the environment as complex ecologies that humans are part of, beyond simple categorizations that might lead microbes be read as ‘good’ or ‘bad’, further challenging the dexterity of ethical frameworks.

## Introducing a queer feminist posthuman framework to justice: thinking with AMR

According to a prominent definition by the WHO (2021), AMR occurs when bacteria, viruses, fungi and parasites change over time and no longer respond to antimicrobials, making infections harder to treat and increasing the risk of disease spread, severe illness and death. However, this dominant understanding of AMR suggests looking at AMR as a human-health issue. Primacy of human health has been criticized by One Health approaches. Yet we see a need for frameworks that can consider more-than-human animals, multispecies perspectives, ecosystems, and stratified human health in the same ethical framework.

When foregrounding a more-than-human approach, the environment must be seen also as key to the development, transmission and spread of AMR to humans, more-than-human animals and plants ( UN Environment Program, 2022, 1). Calibrating a society toward thinking critically about and through AMR resilience and prevention requires attention to species interdependencies ( Brives *et al.*, 2021; Haraway, 2008; Manyau *et al.*, 2022), but must also acknowledge how more-than-human actors already play an important role in environmental and climate politics. Defined in more detail elsewhere ( Sudenkaarne & Butcher, 2024), by *more than*, we not only wish to imply moral significance in the ecocentric and biocentric sense but also to include, in a network theory rather than confessional understanding, spectral realms of ancestors, spirits, ghosts and deities that are crucial actors in several global sense-making systems (cf. De La Cadena, 2010; see also Stengers, 2005). Further, by *more than* we wish to remain open to finding new ways of being with ethics that perhaps could be more attuned to planetary or even cosmological flourishing than current human-oriented approaches. To us, then, *more-than-human* interrogates anthropocentrism in moral significance and ethical analyses but does not wish to abandon or exceed the human *per se*. In this ethical analysis, justice and vulnerability are neighboring concepts: it can be argued, as we do, it is precisely the detection of vulnerabilities that will pave the way to more just worlds.

Pokharel *et al*. (2024) present a set of tensions that hinder efforts to address AMR. They locate these tensions between access and excess of antibiotics in the current population, between personal interests and a wider, shared interest in curbing AMR, between the interests of current populations and the interests of future generations, and lastly, between addressing immediate health threats such as pandemics and AMR as a ‘silent’, chronic threat. To us, this set of tensions reflects the anthropocentric understanding of AMR and does not fathom more-than-human tensions at all. What we suggest is a more-than-human justice orientation in analysis and replacing population thinking with a layered understanding of vulnerability.

A more-than-human justice orientation demands development of new moral theories, which we have argued ( Cañada *et al.*, 2022; Sudenkaarne, 2023; Sudenkaarne & Butcher, 2024) are crucial for AMR in both theory and management. Such a perspective, attuned to both social justice and a more-than-human justice orientation, is crucial to show how intersectional positions within human and more-than-human networks collide with layered vulnerabilities. These vulnerabilities align with existing social justice issues reproduced by race, ability, and gender and sexuality (cf. McKnight, 2022; Sariola *et al.*, 2022; Will, 2018). Further, such a framework should be able to address multispecies vulnerabilities. Vulnerabilities cascade (cf. Luna, 2019) following social-scientific patterns but also take on new layers in AMR. For example, MacPherson and colleagues (2022) have identified specific 'antibiotic vulnerabilities' to highlight how antimicrobial provision in rural Malawi impacts care in conditions of extreme scarcity.

In the framework, posthumanism offers a context, firstly, to situate the dilemma of AMR between human-centered health and a One Health approach. This stance positions the inseparability of the health of humans, non-human animals and the environment as its core premise in a holistic understanding ( Cañada *et al.*, 2022, 1). However, it can be argued One Health is too technoscientific or more of an idea for ethics than a framework. Secondly, posthumanist imaginations for more just futures and presents includes a strong feminist orientation ( Braidotti, 2021).

## Enhancing a queer feminist posthuman framework for (bio)ethics: reflections into vulnerability and care

In order to get to grips with questions of justice and vulnerability across various inequalities calling for a posthuman justice framework, we want to reframe some fundamental questions in relation to AMR. As Sudenkaarne (2023) has suggested, these questions include what kinds of subjects can we imagine as morally significant? How are multispecies relations managed without prioritizing human ways of being? How are intersecting layers of vulnerabilities minimized between groups of people? Is species an oppressive category or in fact needed to ground subversive politics? How to move from human-oriented use of technology to solutions benefitting the entire global ecosystem?

In answering these questions, it is crucial to see how vulnerabilities and justice are integrally connected while still remaining distinct realms of conceptual inquiry. In establishing layers of vulnerability with superwicked problems like AMR ( Levin *et al*., 2012; Littmann *et al*., 2020; Sudenkaarne & Butcher, 2024), visibility is key for both politics and ethics: to acknowledge and address specific needs and injustices that may be in part reproduced through bias in technoscience (cf. van Anders *et al.,* 2023) that requires similar ethical scrutiny as any politics. 

Following Wahlert and Fiester’s (2014) Queer Bioethics Inventory and layered vulnerability established by Luna ( 2009; 2019), The first author of this letter ( Sudenkaarne, 2019; Sudenkaarne, 2021) has developed layers of queer vulnerabilities focusing on intimacy, kinship, agency and ethical sustainability. In bioethical case analysis, for queer and gender sensitive considerations it is important to determine whether medical or other interest is based solely on gender and sexuality variance as curiosity, and does the case reinforce negative perceptions. A crucial comparison is to see whether the analysis or outcome is different for a cis- and heteronormative agent as opposed to a queer one, and in this analysis, does cis- and heteronormativity become a necessary condition for moral coherence. One of the applications of this framework is in thinking about this normativity in the context of drug resistance and gender and sexual variance building on MacPherson and colleagues’ (2022) notion of antibiotic vulnerability. A more detailed engagement offered elsewhere ( Sudenkaarne, in press), MacPherson and colleagues (2022, 2632) conclude that dimensions of care in chronic scarcity present a scenario in which optimizing antibiotic use will require not only stable availability of essential medicines but also a re-ordering of the system around provision of quality care rather than of medicines: thus, practices around care can be understood to both respond to and create antibiotic vulnerabilities. Building on their proposition that considering multiple dimensions of antibiotic vulnerabilities has conceptual promise as it can at once bring to the frame the vulnerability of antibiotics, people and systems ( MacPherson *et al.*, 2022, 2641), for future research is to consider cases of resistance in relation to queer vulnerabilities, such as queer sex workers’ specific vulnerabilities (cf. Manyau *et al.*, 2022) with drug-resistant STI’s (cf. Broom *et al.*, 2023) as situated in specific geographical locations while also informed by global conditions of stratified health, for context-specific programs and specific antibiotic vulnerabilities. Failure to safeguard against such vulnerabilities can lead direct attack on specific lives as rooted in “animus against some of the most marginalized and vulnerable communities” ( Bollinger, 2019).

## Queer feminist posthuman resolutions to a crisis of justice

As AMR ethical dilemmas showcase, considerations about justice have become more and more complex. Dominant theories about justice struggle to sufficiently analyze let alone ethically sustainably alleviate these justice issues as many marginalized stakeholders’ views and vulnerabilities are excluded in a seemingly justice-for-all framework (e.g. Blain, 2020; Tong, 1996). Activist movements such as Black Lives Matter, MeToo and Extinction Rebellion ^ii^ are most recent examples of demonstrating, how presumably shared conditions of living can become eerily uncanny: violence – from physical attacks to symbolic erasure -- haunts everyday activities of some more than others.

Bioethics is an excellent platform for reconfigurations of justice because of its specific engagement with justice through principlism. A highly influential definition of justice as an ethical principle by Tom Beauchamp and James Childress (2019) defines justice as a group of norms to fairly distribute benefits, risks, and costs, linking justice to Rawlsian ideas of distributive justice ( Jecker *et al.*, 2022; Tong, 1996) accepted widely ( Nuffield Council on Bioethics, 2023) and considering justice as some kind of fairness. In global comparison from a moral, societal and political point of view, it is obvious that fairness is not the current state of affairs, not even as distribution of benefits, risks, and costs, let alone in more detailed ethical analysis: with AMR, its costs are the highest in conditions of extreme scarcity ( MacPherson *et al.*, 2022; Nuffield Council on Bioethics, 2023; Sariola *et al.*, 2022). It can be argued that this constitutes the very opposite of fairness ( MacPherson *et al.*, 2022). Moreover, a Rawlsian distributive justice framework does not include multispecies, environmental let alone more-than-human agency, which is a necessary condition for a framework of justice to achieve fairness in any ethically salient way. The notion that justice would be achieved in decision-making, policy-making and ethics-making without critical engagement with the limitations of currently dominant moral theories framing these processes is unfounded: a framework for justice as common morality ( Tong, 1996) might be easily accessible but it leaves out safeguarding against privilege and bias. This often leads to calls for justice “however one conceives of justice” ( Littmann *et al.*, 2020, 441) which, when examining the track record of justice from a queer feminist posthuman vantage point, has in fact not been fair at all (cf. Chao *et al.*, 2022; Lyons, 2023; Pugliese, 2020; Wahlert & Fiester, 2014; Wienhues, 2020; Wolf, 1996).

## Conclusion

To tackle both the false hope and the nihilist despair that fuel injustice, Haraway (2016) has influentially suggested an intellectual method she calls staying with the trouble, making oddkin: that is, increasing human responsibility to marginalized lives and the more-than-human by refusing human exceptionalism through enhanced moral significance. Staying with the trouble requires making oddkin: understanding that “we”, in the more-than-human understanding -- require each other in unexpected collaborations and combinations, “in hot compost piles” ( Haraway, 2016, 4). In an AMR example, this requires subverting the power asymmetries in ethical sense-making for antimicrobial stewardship – reproduction of the neocolonialist division between the global South and North -- understanding intersectional vulnerabilities in access and overuse -- that antibiotics might be the only available treatment in an inadequate basic healthcare context or they can be offered as a choice in more exclusive contexts such occupational health. Most crucially, it requires introducing a relational understanding of health, including more-than-human moral significance while the grunt of posthuman moral responsibility must be born, and better so, by privileged people, us included. A queer feminist posthuman framework aims to facilitate this in a collaborative, ethically sustainable manner.

## Ethics and consent

Ethical approval and consent were not required.

## Data Availability

No data are associated with this article.

## Footnotes

^i ^The first person plural pronouns in the context of this letter refer to Sudenkaarne and Sariola, unless specifically mentioned otherwise.

^ii ^This is an impression of the movement in the Finnish context where the movement explicitly rejects any sympathies to ecofascism.
