# Supplementary material for: Doing ethics with microbes: toward a queer feminist posthuman framework for bioethics
Source: Wellcome Open Res. 2024 Sep 3;9:494. [Version 1] doi: 10.12688/wellcomeopenres.21391.1 (PMC11464968; doi:10.12688/wellcomeopenres.21391.1)
Supplement: Peer review history

# Reviewer report: Reviewer response for version 1

**DOI:** 10.21956/wellcomeopenres.23656.r100024

**Published:** 2024-11-13

**Authors:** Nicolae Morar, Jonathan Beever

**Affiliations:** 1 Environmental Studies and Philosophy, University of Oregon, Eugene, USA; 2 Department of Philosophy, University of Central Florida, Orlando, Florida, USA

Sudenkaarne and Sariola’s open letter contributes to a growing community of thought pushing the boundaries of individuality and moral consider ability; two concepts readily at play in their work if not only implicitly. While we are particularly supportive of such a project that invites us to rethink central concepts in social and political philosophy and in bioethics, we wanted in our review to put forward some challenges that remain unaddressed in their open letter. We certainly share an orientation with the authors to challenge traditional frameworks in bioethics, and the additional attention to our concerns would only strengthen their views.

 First, Sudenkaarne and Sariola articulate the need to follow the historical efforts to dismantle the ontological and ethical categories that have served the oppression of vulnerable populations – human and non-human both and together. The “radical reorientation of human exceptionalism” (4) toward “more-than-human orientations” (ibid) they articulate include remarkably broad networks that focus not only the natural ecologies but also “spectral realms of ancestors, spirits, ghosts, and deities” (5). This effort to overcome *both *the old doctrine of human separability (namely, that humans would be divorced from their environments and should be understood thanks to their inherent properties) *and *the view of human exceptionalism are laudable. They certainly inscribe themselves alongside the effort of a number of environmental ethics thinkers and find an important echo in that literature. However, as we have pointed out elsewhere (Beever & Morar 2016, 2016b, ff.), this ‘contentious’ broadening of moral considerability is only possible with a reorientation of our ontologies, and maybe only then as a result a rethinking of our moral theories. In particular, it is worth noting that while *ontologies *do inform conceptions of normativity (e.g. think here of the importance of ‘individual selves’ for a deontological framework), they do not *generate solely on their own grounds *the content (or the markers) of normativity. The content or the markers of normativity require an additional – ethical – premise, which requires additional explanation and its own grounds for justification. So, to rethink bioethics demands rethinking of what *morally* matters ultimately.

 Second, the authors do voice this tension between ethics and ontology: that limitations in bioethics are in part a result of core metaphysical beliefs core to our Western worldview, including the doctrine of separability (Beever & Morar forthcoming) and the doctrine of exceptionalism (Gruen 2003). Yet thinking more-than-human while at the same time not abandoning or exceeding the human *per se* (5) and “seeking to keep existing social justice issues between groups of people in the same framework” (4) is contraindicative if not contradictory. Once we endorse a microbial view of life, including of human organisms and of their intrinsic porosity, and we decide to let go of central categories of ‘individuality and selves’, one would need to recognize immediately the ‘danger’ of dissolving the very taxonomy that has produced our moral protections. It could be the case that thinking with microbes invites us to question the very notion of ‘autonomy,’ since there are no such things as individual-atomistic-moral agents. But, one cannot engage in this effort while only alluding and not taking seriously the dark side to the interdependence (Beever 2020, Beever 2022) as the history of bioethics has shown us.

 Third, their ideas connote a world as being one built of interconnections ( *our term*), including their justices and vulnerabilities (5). Through their discussion of the COVID-19 pandemic and references to the One Health paradigm, they point to the urgency of finding “new ways of living…with each other, with other animals, plants, and our microbial ancestors” (4). Our earlier work on bioethics, public health ethics, and microbial life specifically articulated the centrality of relationality to rethinking bioethics, arguing that the science of microbial ecology identifies spaces between interconnection and interdependence or the distinction between being shaped by ecological relations and being made up of them (Morar & Bohannan 2019, Beever & Morar 2018, 2016, 2016b, Beever & Whitehouse 2017). More importantly, *what morally matters *implies identifying *either *novel units of care *or* *new ecologies/ networks *of relationality that have a moral binding on us. If microbes teach us that we have wrongly drawn the contours of ‘individuality’ or that we have overly focused on individuals at the expense of communities or ecological networks, it still behooves us to provide an argument as to *how *‘doing ethics with microbes’ deals with new contours for moral subjects or with new forms of relationality.

 Indeed, there are many ways in which we have been working as strong allies in arguing and generating further support for Sudenkaarne and Sariola’s open letter. In contrast, we have always tried to separate the *declarative *from the *critical reasoned, *with the hopes that one understands both the important challenges that a microbial view raises, while not overestimating their input or their normative force. The amazing work of Maureen O’Malley (2014), especially in its critical form of the hype around microbiome research, stands as a litmus test to any future argument that assumes to easily a definitive entanglement of the descriptive and of the normative. Certainly, we do believe that notions of the ‘self’, ‘individual’, ‘organism’, ‘species’, ‘genotype’, etc. are fully called into question but we are weary of reading too rapidly a loaded normative response whose positive input would be read off this new world view.

 Probably, this is the place at which we part ways with the authors, given the ways in which their open letter almost too easily blends descriptive flavoring with prescriptive intentions, while not sufficiently arguing for the *specific *reasons that are required for each statement (of fact or of values). As David Hume would say, one cannot derive an ought from an is, so the challenge remains that *once *we alter our metaphysical views, can we shift gear to *bioethics *while being mindful of not committing some kind of fallacy?

 For this reason, in order to avoid potential missteps in this (and in any future) line of argument, we believe that such work requires:

 1. Reevaluating the importance of the interconnectedness-interdependence distinction, and

 2. Articulating a novel position within the amalgam of animal, environmental, and posthuman feminist ethics that genuinely question the nature of exceptionalism, not only in its anthropocentric form, but also within the proposed ‘vulnerability’ framework. While we are not convinced those steps demand a new moral theory(ies) as the authors claim (Sudenkaarne 2023, Sudenkaarne & Butcher 2024), we truly welcome their effort. In our view, we might rather argue for a shift in metaethics – the nature of the good and right, and to whom or what it applies. Indeed, this has been the work for decades of the feminist, post humanist, and queer scholars they cite in and around animal ethics, feminist ethics, and the radical ontologies with which they engage. While critical, far from us to give up on what Sudenkaarne and Sariola do offer us an enticing normative recipe here.

Does the article adequately reference differing views and opinions?

Yes

Are all factual statements correct, and are statements and arguments made adequately supported by citations?

Yes

Is the Open Letter written in accessible language?

Yes

Where applicable, are recommendations and next steps explained clearly for others to follow?

Yes

Is the rationale for the Open Letter provided in sufficient detail?

Yes

Reviewer Expertise:

bioethics, philosophy of microbiology

We confirm that we have read this submission and believe that we have an appropriate level of expertise to confirm that it is of an acceptable scientific standard.

## References

[1] Philosophy, Film, and the Dark Side of Interdependence; Beever, J. (Ed). *Rowman & Littlefield; Lexington Books* .2020;

[2] The Horror of Relations; Beever, J. *Philosophy Now* .2022;volume 152; Reference source

[3] When the Outside Becomes Inside: Causal and Constitutive Environmental Relations in Bioethics; Beever, J. & Morar, N; (Ed. C. Richie); forthcoming. *Oxford Handbook of Environmental Bioethics* .

[4] Bioethics and the Challenge of the Ecological Individual; Beever, J. & Morar, N. *Environmental Philosophy* .2016.;13(2):215-238;

[5] The Porosity of Autonomy: Social and Biological Constitution of the Individual in Biomedicine; Beever, J&Morar, N. *American Journal of Bioethics* .2016;16(2): 34-45;10.1080/15265161.2015.112079326832092

[6] The Epistemic and Ethical Onus of ‘One Health’; Beever, J. & Morar, N. *Bioethics* .2018,33(1): 185-194;10.1111/bioe.1252230341916

[7] The Ecosystem of Bioethics: Building Bridges to Public Health; Beever, J. & Whitehouse, P.J. *Jahr: European Journal of Bioethics* .2017;8/2(16): 227-243;

[8] The Moral Status of Animals;Gruen, L. *Stanford Encyclopedia of Philosophy* . Reference source

[9] Conceptual Ecology of the Human Microbiome; Morar, N. & Bohannan, B.J.M. *Quarterly Review of Biology* .. 2019;94:2; 10.1086/703582

[10] : Philosophy of Microbiology.2014; 10.1017/CBO9781139162524 10.1017/CBO9781139162524

[11] : From super-wicked problems to more-than-human justice: new bioethical frameworks for antimicrobial resistance and climate emergency. *Monash Bioeth Rev* .2024; 10.1007/s40592-024-00197-z 10.1007/s40592-024-00197-z 38990507 PMC11850483

[12] Toward a queer, feminist, posthuman analysis of AMR and bioethics; Sudenkaarne T. *The Polyphony* .2023; Reference source

# Reviewer report: Reviewer response for version 1

**DOI:** 10.21956/wellcomeopenres.23656.r101073

**Published:** 2024-10-09

**Authors:** Margrit Shildrick

**Affiliations:** 1 Stockholm University, Stockholm, Sweden

As a biophilosopher and posthumanist bioethicist with an abiding interest in microbial life, I read the Open Letter with great interest. I already know something of the work of CSSM and welcome the initiatives that they have developed. I’m not entirely sure of the status of an Open Letter or of its intended audience but I hope my comments will be of some use.

 The authors set an ambitious aim of broadening the reach of bioethics to take into consideration the more-than-human by developing a posthumanist, queer and feminist critique that might rewrite the terms of justice. Part of their intention is to focus on the concept of layered vulnerability, which is a good starting point, and to bring that approach to the consideration AMR in particular, where injustice and differential vulnerabilities are plain to see. A secondary aim is give a sense of the wider work of CSSM, although this gets very little play after the introduction. The couple of references to slime mould were almost superfluous without further explanation.

 Right away I want to say that there is a lot of potential in exploring this area and I hope that the authors will not be disheartened by my reservations. One of the most meaningful questions for me was how to elucidate an ongoing social justice between people in the same frame as rejecting the anthropocentrism that that implies. Perhaps the authors might try to think of it in successive registers (even layers?) rather than trying to come up with an all inclusive framework. I was somewhat alarmed right near the beginning when the conceptual analysis promised to bring together ‘bioethical principalism, analytical philosophy, feminist thought and queer theory’, and ‘posthuman framework for justice’. I struggle to see how principalism and analytic philosophy are really going to work in that context. In my understanding the whole point of posthumanism is to provide a critique of those approaches which rest of predetermined structures and rational analysis. It would be excellent if the authors could pull this off, but I fear it may be an impossibility. Microbial life simply doesn’t work like that.

 My feeling is that the authors are attempting to use philosophy in a way that simply highlights the redundancy of normative forms rather than opens up new ways of thinking. If we want to take more-than-human life seriously, it seems important not to rely on fully humanist modes of analysis. There is certainly a paradigm shift to be made but it’s not really happening when slime mould is characterised as being without a central order – an important observation - ‘but not without leadership and responsibility’. What could this possibly mean?  On the other hand, I do like the idea of a more-than human justice orientation which replaces population thinking with a layered understanding of vulnerability. The problem is that going back to Rawls (not to mention those old favourites Beauchamp and Childress) for example to underpin what is meant by justice rather vitiates the effect. I’d hope that an adequate posthumanist justice was far more flexible.

 I don’t know how much it matters for a project that is not primarily aimed at philosophers, but I’d also welcome some distinction being made between ethics and morals, rather than using the 2 terms interchangeably. The same lack of definition means that the claim to a queer perspective is never really explained, although I agree with the authors that it could be a very appropriate way of reconfiguring how we think about microbial life.

 The question: ‘is species an oppressive category or in fact needed to ground subversive politics’ is intriguing’ and one I hope the authors will constantly ask themselves and their readers. The dimensions of AMR are a wonderful platform for taking this further. Although it looks at microbes in a very different context, the authors might be interested in looking at a couple of my own articles on viruses.

- Entangling Viral Encounters, Immunity and the Gift’, *Interconnections: Journal of Posthumanism *(2022) 1, 2 *: *57-72.
- COVID-19:n biofilosofiaa ja-politiikkaa: Pandemian uudelleenarviointi Roberto Espositon immuniteettiparadigman valossa’, Finnish text only in *Niin & näin* (2022) 2: 87-95. Online at https://netn.fi/sites/www.netn.fi/files/netn222-18.pdf (This Finnish version is shorter but deals with similar issues).

One final point: the Open Letter is very hard to read due to the grammar mistakes which sometimes obscured what point was being made. When the authors take this further in terms of publications, I’d recommend some serious language editing.

Does the article adequately reference differing views and opinions?

Partly

Are all factual statements correct, and are statements and arguments made adequately supported by citations?

Yes

Is the Open Letter written in accessible language?

Partly

Where applicable, are recommendations and next steps explained clearly for others to follow?

Partly

Is the rationale for the Open Letter provided in sufficient detail?

Partly

Reviewer Expertise:

biophilosophy and posthumanist bioethics with a current focus on microbial life

I confirm that I have read this submission and believe that I have an appropriate level of expertise to confirm that it is of an acceptable scientific standard, however I have significant reservations, as outlined above.

## References

[1] Entangling Viral Encounters, Immunity and the Gift. *Interconnections: Journal of Posthumanism* .(2022) 1, 2: 57-72.;

[2] COVID-19:n biofilosofiaa ja-politiikkaa: Pandemian uudelleenarviointi Roberto Espositon immuniteettiparadigman valossa. *Niin & Näin* .(2022) 2: 87-95; Reference source

# Reviewer report: Reviewer response for version 1

**DOI:** 10.21956/wellcomeopenres.23656.r101067

**Published:** 2024-10-09

**Authors:** Christopher Mayes

**Affiliations:** 1 Deakin University, Waurn Ponds, Australia

The letter aims to bring social scientific work on microbes into conversation with applied ethics/moral theory, especially a more expansive understanding of bioethics that includes the socio-political-environmental situatedness of individuals and health. 

 The letter builds on Sudenkaarne’s conference presentation at the Oxford Global Health and Bioethics 2023 - which I did not attend and have not read, but used AMR to show the limits of bioethical thinking and to argue for the importance of "queer feminist posthuman framework". 

 In this letter Sudenkaarne and Sariola aim to expand/build on their previous approach by developing a more-than-human-justice approach. The authors make a clear and compelling case that a "more-than-human justice orientation demands development of new moral theories". This is particularly the case in bioethics where there is an overreliance on narrow Anglo-American (aka Rawlsian) conceptions of justice, as the authors point out.

 I am hesitant to point to my own work in a review, but there was a recent target article by Keisha Ray and Jane Cooper in the AJOB and subsequent responses that touches on some of the limitations of current bioethical conceptions of justice in relation to the environment and more-than-human world that the authors discuss. The response I co-wrote with Nicolae Morar engages with some of the limitations of utilising Beauchamp and Childress's anthropocentric conception of justice to attend to environmental and microbial injustices. Mayes et.al (2024 ^1^ )

Does the article adequately reference differing views and opinions?

Yes

Are all factual statements correct, and are statements and arguments made adequately supported by citations?

Yes

Is the Open Letter written in accessible language?

Yes

Where applicable, are recommendations and next steps explained clearly for others to follow?

Yes

Is the rationale for the Open Letter provided in sufficient detail?

Yes

Reviewer Expertise:

moral philosophy, bioethics, social theory.

I confirm that I have read this submission and believe that I have an appropriate level of expertise to confirm that it is of an acceptable scientific standard.

## References

[1] : Environmental Injustices within Us: The Case of the Human Microbiome and the Need for More Creative Bioethics. *Am J Bioeth* .2024;24(3) : 10.1080/15265161.2024.2303135 67-70 10.1080/15265161.2024.2303135 38394022

# Reviewer report: Reviewer response for version 1

**DOI:** 10.21956/wellcomeopenres.23656.r100019

**Published:** 2024-10-08

**Authors:** Aaron Bradshaw

**Affiliations:** 1 Humboldt University of Berlin, Berlin, Germany

**Overview/summary**

 This Open Letter has two key themes: 1) an introduction to the Wellcome-funded Centre for the Social Study of Microbes (CSSM) at the University of Helsinki in Finland, and 2) an argument for a posthuman, queer, and feminist (bio)ethics. The second theme is most clearly spelled out in the author’s Plain Language Summary: “[This] queer feminist posthuman framework for bioethics offers a platform to consider injustices caused by human action to environments and more-than-human animals and injustices between groups of people in the same framework.” Queer, feminist, and posthuman is therefore offered as an analytical triad that, when brought into conversation with normative bioethics, can more adequately address injustices and vulnerabilities that are disproportionately experienced by people based on the categories of gender and sexuality, and between the human/nonhuman divide. There are important connections (or intersections) between these categories which the Letter also touches on. 

 In situating their bioethical framework in the context of human-microbial entanglements, the authors note that the complex relations between humans, microbes, animals, and environments “require new theoretical and methodological approaches.” They identify antimicrobial resistance (AMR) as a particularly urgent manifestation of this: “In this letter, the focus is on the need for a new framework and the structure of this framework to grapple with ethical questions of AMR.” Hence, this framework is both urged by the socio-material reality and complexity of AMR and is offered as an approach to address it on an ethical basis. Specifically, AMR is identified as a dilemma for which current ethical frameworks based on liability, utilitarian, and fundamentally human-centric approaches are sorely lacking. The situation created by humanist and masculinist ethics is framed as a “crisis of justice”, a claim backed up by reference to how the brunt of AMR is unevenly distributed and disproportionately experienced by poor, racialized, and queer groups. To this, and as this Open Letter argues, we might consider other-than-human organisms, multispecies networks, and geophysical environments as subjects worthy of moral consideration, as relational systems both affecting and affected by AMR. So far, animals and environments have generally been understood solely as vectors and reservoirs for AMR (Helliwell et al., 2020), and not as possible subjects of (in)justice, even within a One Health framework (see Cañada et al., 2022). The exploitation of other-than-human agents and physical environments tends to go hand in hand with the oppression and marginalisation of certain human groups (and also the construction of those groups as such). Both follow logically from a moral theory that is centred on the (white, male, Western) human subject, a hierarchical vision of human and nonhuman life, and an ontology of separate, bounded, and autonomous entities. A framework that includes marginalised groups of humans and non-humans - what we might call ‘more-than-human intersectionality’ - in the same moral landscape therefore seems to be both a necessary outcome of posthuman and feminist ontologies as well as its optimistic horizon.

 I would like to point out that my expertise does not lie within queer theory, intersectional theory, or feminist theory. Nor am I a bioethicist. However, I have research interests in the social study of microbes, socio-microbial relations, and in developing new interdisciplinary methods, models, and theories for engaging with microbial communities and ecologies. As such, this Letter is of scholarly/research interest to me and it is great to see so many different approaches being applied to our understanding of human-microbial relations. Even more great is the aim to translate these theories into practical applications with possible policy implications on the horizon. 

**Suggestions for revision**

 Although I am highly sympathetic to and supportive of the authors’ scholarly and ethical aims, my main concern is that it is challenging to follow the development of the argument and its overall conclusions as presented here. This could partly be a reflection of my expertise and of different authors’ writing styles, but I also think that many of the sentences are structurally/grammatically complex and contain highly technical language that isn’t properly introduced/explained. A closer attention to the research context is also necessary. On this basis, I think this Letter could be significantly improved by attention to three key areas: 1) Clearer statement of the aims; 2) Clarification and integration of key concepts and the development of the argument; and 3) Contextualisation of the this specific piece of work within the much wider field of microbe studies (and the work of the CSSM). Although what I have written below is relatively extensive and may seem critical, I think that attention to these details could draw out the excellent arguments that are at points somewhat buried in the piece. 

**1) Introduction, aims, and context**

 From the introduction two (or maybe three) explicit aims are identified:

- To introduce both a theoretical and practical initiative that seeks to further ground solidarity between justice movements while simultaneously promoting a more-than-human-justice orientation for cosmopolitical flourishing;
- Offer this framework as relevant to a broader bioethical, social scientists working on human-microbial relations [ *sic*], and philosophical research community; 
- (and for grounding policy).

And another implicit aim: 

- To introduce the CSSM as the context for this research and a hub for interdisciplinary social scientific research into human-microbial relations.

To adequately address four aims in such a short article is a tall order. Moreover, the first aim is formulated in complex language and deploys technical concepts that have rich scholarly genealogies that would require explicit introduction/discussion (such as ‘cosmopolitical flourishing’). In that sense, the article would benefit from a clearer articulation of the aims. I would suggest distilling the aim(s) in simple, straightforward language. What do the authors want us, as readers, to take away as the key aims of the letter?  

 Secondly, the given rationale for the letter is to “discuss social scientific work with microbes from an applied ethics and moral theoretical point of view, which is a part of a philosophical approach to bioethics.” Yet, the specific focus in this article is AMR and not social scientific with microbes in general. This subsumes a significant amount of diversity with the field of microbe studies (see points below). The rationale appears to be more specific than what is offered here (for instance, the failure of anthropocentric ethical frameworks in responding to the more-than-human and intersectional injustices creating and created by AMR) and therefore needs spelling out more clearly. 

 In introducing their (bio)ethical framework, the authors draw heavily on Sudenkaarne and Butcher’s recent (2024) publication in Monash Bioethics Review where the framework is developed in greater detail.* This research was also presented at the Oxford Global Health and Bioethics conference and is discussed in other publications and blogs. In that context, I would encourage the authors to tease out what specific contribution this Letter makes in addition to these already published works. I am not suggesting there is not one, and I understand that the contribution is related to the format of an Open Letter as a forum for “discussing policies relevant to a broad research community, presenting guidelines or white papers, or announcing new initiatives” (Wellcome Open Research, n.d.). That particular orientation needs to be put more up-front. 

 Moreover, I would also suggest that the introduction could be used to more tightly and precisely convey the central concepts, overall argument, conclusions, and future directions offered in this Letter (see point below).

^* The following sentence should be in quotation marks, as it is lifted from Sudenkaarne and Butcher, 2024: “by more than, we not only wish to imply moral significance in the ecocentric and biocentric sense but also to include, in a network theory rather than confessional understanding, spectral realms of ancestors, spirits, ghosts and deities that are crucial actors in several global sense-making systems.” As does the following sentence: “what kinds of subjects can we imagine as morally significant? How are multispecies relations managed without prioritizing human ways of being? How are intersecting layers of vulnerabilities minimized between groups of people? Is species an oppressive category or in fact needed to ground subversive politics? How to move from human-oriented use of technology to solutions benefitting the entire global ecosystem?”^

**2) Key concepts and development of the argument**

 The arguments being made in this article are quite complex and draw on a wide range of scholarly traditions. This might be considered one of the article’s key strengths as well as one of its key weaknesses. It is challenging to integrate concepts from analytical philosophy/bioethics, queer studies, microbial ecology, global health and posthuman thought into a coherent whole. Although the authors have made good progress in that direction, being more precise and less assuming on the part of the reader would improve the article significantly. 

 Important concepts from bioethics such as ‘vulnerability’ and ‘justice’, whilst critical for grounding the article’s aims and arguments, are not clearly introduced at the right time. Although this might be less of a concern for a specialised readership, the technical meanings of these concepts - and particularly how they are being used in this piece - might be lost on researchers from other fields who would otherwise be interested in the more-than-human dimensions of AMR. As someone who doesn’t have a background in philosophy or ethics, I found myself having to dig deep to understand some of the arguments being made. For example, what does it mean to ‘establish/develop layers of vulnerability’? How would this apply to nonhuman life? Similarly, ‘justice’ is defined/introduced but not until the last section whilst it is conceptually leveraged in earlier sections.

 Another important concept is ‘framework’ or ‘framework-thinking’. The definition, scope, and value of a ‘framework’ are implied by the article but this is not made explicit. For instance, how do the concepts of justice and vulnerability fit into the framework being offered here? What is the point of a framework with respect to normative ethics? Is the idea that it can be used to make decisions based on a particular scenario in an almost formulaic way, or is it more fluid? Is the framework offered here a modulation of bioethical principlism, in which the principles are extended through the intersectional and more-than-human dimensions, or does its articulation critique the (anthropocentric) ground of this framework and offer a more radical reorientation?

 One suggestion might be to introduce/discuss these concepts early on. What are the stakes in terms of AMR? *Why *a feminist, queer, and posthuman framework? What makes it feminist, queer, and posthuman in an explicit sense? What do current frameworks lack with respect to addressing vulnerability and justice (of specific groups and lifeforms)? How does the new framework address this in practice? In sum, what is the overall argument? I would suggest there is too much assumption on the part of the reader about the state of AMR, its ethical landscapes, and what the key issues are. Even in the plain language summary we learn that “the complex relations between microbes…require new ways to think about these issues and aims to resolve them.” But why do they? What are the issues being responded to here? By working on the structure of the article and really focusing to clarify the introduction, the answers to these other questions should fall into place.

 Finally, given that this is an Open Letter, it would be great to see some clear examples of where the proposed framework might be applied and what the outcomes might be (i.e., where it might guide practice down different routes). This is touched on in the last section, but again it assumes a lot on the reader’s part. The strong emphasis on 'more-than-human' and 'multispecies' also suggests a need to provide examples that include other-than-human actors and their flourishing (the current example is focused on humans). If this ethical framework is to guide policy at different levels of health, environment, and ecology, then these examples/empirical cases need to be articulated in more simple and direct language. 

**3) CSSM and the field of microbe studies**

 An introduction to, and discussion of, the CSSM is an important component of this Letter, particularly as a Wellcome funded research hub and the environment for the development of the research. However, there is something of a disconnect between the Letter’s formulation of a relatively specific bioethical framework for AMR and the wider research environment of the CSSM. This is most evident in the plain language summary where the logical connection between the two is simply that the former “has been inspired by the collective work” at the latter. This is also evident in the main abstract and second section in the main body where points about sensory approaches, ethnography, fermentation, urban gardens etc. feel awkward and poorly integrated with what is primarily on offer here. The discussion of the CSSM’s work is relatively generic and doesn’t contain much more analysis than what we can learn from the Hub’s website. The research environment of the CSSM appears to be very vibrant and I would encourage the discussion here to do more justice to that. 

 There also seems to be something more specific about the relationship between the CSSM and the ethics developed here that can be teased out more directly in this section. I would almost like to suggest that the CSSM itself could be seen as a testing ground not only for the theoretical development of the framework discussed in the article, but also for its practical application. Indeed, this is implicit in the CSSM sections’ last paragraph, but here the work of the CSSM is discussed in relation to other concepts (e.g., unruliness) and theories (flat ontology) although we do see a reference to ‘practical ethics’ as well. In a similar way, the metaphor of the slime mold as a model for the organisation of the CSSM is provocative but is not well-developed here. Although a more thorough analysis is referenced, it is difficult to fully grasp what is being offered. For instance, the biological and micro-scalar mechanisms of microbiomes also provide fruitful metaphors for exploring ethics, relationality, and sociality (for e.g., see Young, 2020). So why is the slime mold being upheld as an exemplar of non-hierarchical organisation, rather than these other processes? What is it about microbial agency specifically, that poses a challenge to practical ethics? I can sense a link between the “non-hierarchical” organisation of the CSSM, the article’s model of a posthuman ethics, and something about the complexity of how microbes are affecting and being affected by ‘human’ worlds. Yet, this link remains somewhat implicit and could be drawn out more. Doing so will benefit the coherence of the article and the integration of its different aims/rationales.

 Finally, the field of microbe studies is diverse and spans a significant range of disciplinary formations concerned with various relations, environments, forms of governance etc. The article does not sufficiently engage with the wider context of microbe studies. The social study of microbes - *named as such* - is an emerging field of interdisciplinary inquiry, but the field has important precursors and contemporaneous strands in, for example, medical history (Luckin, 1977; Hardy 1984), environmental history (Clifford, 2016), urban theory (e.g., Gandy, 2006, 2023), feminist STS (Hird, 2010; see Hird’s other work, too), human geography (e.g., Lorimer et al., 2020) social theory (Voelkner, 2019, Ironstone, 2020), multispecies studies (Lorimer, 2016) and posthuman theory (Herbrechter, 2018 Hird and Yusoff, 2016; see also the table in my recent publication, Bradshaw, 2024). This is only a fraction of the social scientific research into microorganisms and human-microbial relations. Hence, there is a concern that the diversity of microbe studies is potentially obscured in this article because of its focus on antimicrobial resistance and pathogenic microorganisms. I am not suggesting that the authors attempt a review of the field (which would be impossible) but acknowledge its existence. It is always challenging to demarcate a field of inquiry based on subject matter alone, especially when so many disciplines have a stake in it.

Does the article adequately reference differing views and opinions?

Partly

Are all factual statements correct, and are statements and arguments made adequately supported by citations?

Partly

Is the Open Letter written in accessible language?

Partly

Where applicable, are recommendations and next steps explained clearly for others to follow?

Partly

Is the rationale for the Open Letter provided in sufficient detail?

Partly

Reviewer Expertise:

Human-microbial relations; more-than-human geography; microbial evolution; anthropogenic environments; urban environments; pollution; interdisciplinary methods; visual methods.

I confirm that I have read this submission and believe that I have an appropriate level of expertise to confirm that it is of an acceptable scientific standard, however I have significant reservations, as outlined above.

## References

[1] : The invisible city: The mundane biogeographies of urban microbial ecologies. *Geo: Geography and Environment* .2024;11(2) : 10.1002/geo2.148 10.1002/geo2.148

[2] West ham and the river lea: A social and environmental history of londons industrialized marshland; Clifford J.(2017);1839–1914; Vancouver BC; University of British Columbia Press;

[3] The bacteriological city and its discontents; Gandy, M. *Historical Geography* .(2006);34(6);14–25.;

[4] : Zoonotic urbanisation: multispecies urbanism and the rescaling of urban epidemiology. *Urban Studies* .2023;60(13) : 10.1177/00420980231154802 2529-2549 10.1177/00420980231154802

[5] : Abundance in the Anthropocene. *The Sociological Review* .2019;67(2) : 10.1177/0038026119830907 357-373 10.1177/0038026119830907

[6] : Water and the search for public health in London in the eighteenth and nineteenth centuries. *Med Hist* .1984;28(3) : 10.1017/s0025727300035936 250-82 10.1017/s0025727300035936 6390024 PMC1139446

[7] : Environmental imaginaries and the environmental sciences of antimicrobial resistance. *Environment and Planning E: Nature and Space* .2021;4(4) : 10.1177/2514848620950752 1346-1368 10.1177/2514848620950752

[8] : 23 Microbes.2018; 10.1515/9781474418423-026 354-366 10.1515/9781474418423-026

[9] : Postcolonial Global Health, Post-Colony Microbes and Antimicrobial Resistance. *Theory, Culture & Society* .2022;39(3) : 10.1177/0263276420981606 145-168 10.1177/0263276420981606

[10] Subtending relations: Bacteria, geology, and the possible. Genealogies of Speculation: Materialism and Subjectivity Since Structuralism; Hird, M. J;Yusoff, K.(2016);319-342;

[11] : Meeting with the Microcosmos. *Environment and Planning D: Society and Space* .2010;28(1) : 10.1068/d2706wsc 36-39 10.1068/d2706wsc

[12] : Me, my self, and the multitude: Microbiopolitics of the human microbiome. *European Journal of Social Theory* .2019;22(3) : 10.1177/1368431018811330 325-341 10.1177/1368431018811330

[13] : Making the microbiome public: Participatory experiments with DNA sequencing in domestic kitchens. *Transactions of the Institute of British Geographers* .2019;44(3) : 10.1111/tran.12289 524-541 10.1111/tran.12289

[14] : Gut Buddies. *Environmental Humanities* .2016;8(1) : 10.1215/22011919-3527722 57-76 10.1215/22011919-3527722

[15] : The final catastrophe—cholera in London, 1866. *Medical History* .1977;21(1) : 10.1017/S0025727300037157 32-42 10.1017/S0025727300037157 PMC1081893320408

[16] : From super-wicked problems to more-than-human justice: new bioethical frameworks for antimicrobial resistance and climate emergency. *Monash Bioeth Rev* .2024; 10.1007/s40592-024-00197-z 10.1007/s40592-024-00197-z 38990507 PMC11850483

[17] : Riding the Shi: From Infection Barriers to the Microbial City. *Int Political Sociol* .2019;13(4) : 10.1093/ips/olz016 375-391 10.1093/ips/olz016 34191935 PMC7149467

[18] *Wellcome Open Research;author guidelines;Open Letter* . Reference source

[19] Anthropology of health environments beyond determinism:Tribes without Rulers: Bacteria Life in the Human Holobiont Seeberg J; Roepstorff A; Meinert L. *Biosocial Worlds* .Young A;2020 (pp. 109-123). University College London;
